# Longitudinal Trajectories and Psychosocial Predictors of Postpartum Sexual Dysfunction from Early Pregnancy to 12 Months Postpartum

**DOI:** 10.3390/medicina62030541

**Published:** 2026-03-14

**Authors:** Aris Boarta, Adrian Gluhovschi, Marius Lucian Craina, Carmen Ioana Marta, Bogdan Dumitriu, Ioana Denisa Socol, Madalina Ioana Sorop, Bogdan Sorop

**Affiliations:** 1 Doctoral School, Faculty of Medicine, Victor Babes University of Medicine and Pharmacy, 300041 Timisoara, Romania; aris.boarta@umft.ro (A.B.); bogdan.dumitriu@umft.ro (B.D.); ioana.socol@umft.ro (I.D.S.); madalina.pop@umft.ro (M.I.S.); 2 Department of Obstetrics and Gynecology, Victor Babes University of Medicine and Pharmacy, 300041 Timisoara, Romania; mariuscraina@umft.ro (M.L.C.); carmen.marta@umft.ro (C.I.M.); bogdan.sorop@umft.ro (B.S.)

**Keywords:** sexual dysfunction, physiological, pregnancy, postpartum period, depressive disorder, body image

## Abstract

*Background and Objectives:* Pregnancy and the postpartum period profoundly affect female sexual function, yet longitudinal data integrating obstetric and psychosocial domains are scarce. We aimed to chart sexual-function trajectories from early pregnancy to 12 months postpartum and identify predictors of persistent dysfunction. *Materials and Methods:* In this single-center prospective cohort, 187 pregnant women were eligible to complete the FSFI at three trimesters and at 6–8 weeks, 3 months, and 6–12 months postpartum, plus postpartum PHQ-9, WHOQOL-BREF, and body-image scales. Associations with FSFI-defined dysfunction (FSFI < 26.55) and continuous FSFI were examined, of which 90 women were included for having documented dysfunction. *Results:* Mean FSFI declined from 27.4 ± 3.9 (first trimester) to a nadir of 20.1 ± 4.2 at 6–8 weeks postpartum, with partial recovery to 25.5 ± 4.0 at 6–12 months (*p* < 0.001). Depressive symptoms were higher in women with dysfunction (PHQ-9 8.8 ± 3.3 vs. 6.7 ± 3.5; *p* < 0.001) and correlated inversely with FSFI (r = −0.39; *p* < 0.001). A multivariable model explained 19% of FSFI variance, with each 1 SD PHQ-9 increase predicting a 1.2-point FSFI decrease (*p* = 0.005). Body-image disturbance exerted a partially PHQ-9-mediated effect, and three FSFI trajectory clusters showed postpartum dysfunction rates from 28.6% to 89.7%. A combined psychosocial prediction model achieved an AUC of 0.9 with a sensitivity and specificity of 0.8. *Conclusions:* Postpartum sexual dysfunction was common and persisted in many women at one year; depressive symptoms, body image, and psychological quality of life were more influential than mode of birth, breastfeeding, or pelvic-floor symptoms.

## 1. Introduction

Female sexual function is a key component of quality of life, yet pregnancy and the puerperium represent times when sexual health is particularly vulnerable. Hormonal shifts, weight gain, bodily changes, and evolving couple dynamics converge on desire, arousal, lubrication, orgasm, satisfaction, and pain. Many women report reduced sexual frequency and satisfaction, and a substantial proportion meet criteria for sexual dysfunction during late gestation or after childbirth, with recent reviews estimating a prevalence between 40% and 80% depending on timing and instrument used [1,2,3]. Across cohorts from Europe, North America, and the Middle East, women frequently describe pregnancy and the first postpartum year as periods of transition in which sexual concerns are common but rarely discussed with clinicians [1,2].

During pregnancy, hormonal fluctuations, increasing uterine size, and local vascular changes may alter genital responsiveness and comfort. Prospective and cross-sectional FSFI-based studies show that desire, arousal, and orgasm scores often decline progressively from the first to the third trimester, while pain and lubrication problems become more frequent [2,4,5]. After delivery, the early postpartum period is marked by perineal healing, lochia, lactational hypoestrogenism, and sleep fragmentation; dyspareunia, vaginal dryness, and low desire are particularly frequent in the first 3–6 months postpartum, and in many series, sexual function does not fully return to pre-pregnancy levels by 6–12 months [1,4,5,6].

Obstetric events can further influence sexual outcomes. Instrumental vaginal births, higher degrees of perineal trauma, and complicated episiotomies are associated in some, though not all, studies with more intense perineal pain, pelvic-floor symptoms, and lower FSFI scores in the first postpartum year [7,8,9]. Dyspareunia rates of 20–60% at 3–6 months postpartum have been reported, with severity related to perineal pain, scar tenderness, and pelvic-floor dysfunction as well as to psychosocial distress [6,7,8]. Cesarean delivery, while avoiding perineal injury, introduces its own set of scars, recovery demands, and pelvic-floor sequelae, and recent meta-analytic data suggest that differences in long-term dyspareunia between cesarean and vaginal birth may be smaller than previously assumed [6,9].

Breastfeeding is another key factor. Lactational amenorrhea, hypoestrogenism, fatigue, and the practical demands of infant care can all modulate sexual interest and response, yet findings on whether exclusive breastfeeding aggravates or protects against dysfunction remain mixed. Longitudinal and cross-sectional work indicates that breastfeeding, especially when combined with persistent perineal pain or low partner support, is associated with lower desire and more dyspareunia at 6–12 months postpartum in some cohorts [6,7,10]. A recent systematic review and meta-analysis focusing specifically on breastfeeding women highlighted substantial heterogeneity in effect sizes but confirmed that sexual dysfunction is common in this group and under-recognized in routine care [10].

Psychosocial determinants—particularly depressive and anxiety symptoms, body-image concerns, and perceived quality of life—are increasingly recognized as central to perinatal sexual health. Studies in pregnant and postpartum populations show that worse mental health scores and lower satisfaction with one’s changing body are consistently linked to poorer sexual function and lower sexual satisfaction [11,12,13]. Pregnant women who report higher stress, depressive symptoms, or poorer environmental and psychological quality-of-life scores have lower sexual satisfaction and more pain, while postpartum cohorts with low self-esteem or negative body image describe more avoidance of sexual activity and greater distress about sexual changes [11,12,13].

Despite this growing literature, important gaps remain. Most existing studies are cross-sectional, restricted to either pregnancy or postpartum, or use heterogeneous measurement tools, making it difficult to compare trajectories over time or to integrate obstetric, breastfeeding, pelvic floor, and psychosocial domains in a single model [3,5,6]. Instruments such as the WHOQOL-BREF and the FSFI provide standardized assessments of quality of life and multidimensional sexual function, but many studies employ abbreviated or non-validated scales, or report only total scores without exploring domain-specific patterns [5,14,15,16]. Compared with previous longitudinal cohorts, our study combines dense FSFI sampling from early pregnancy through 12 months postpartum with concurrent assessment of depressive symptoms, generic quality of life, and body-image disturbance in an Eastern European context, allowing us to integrate obstetric and psychosocial domains within a single longitudinal and predictive framework.

In this context, we conducted a single-center prospective cohort study in Romania with three primary aims: (1) to quantify sexual function longitudinally across all three trimesters and up to 12 months postpartum using the FSFI; (2) to compare the relative contributions of obstetric, breastfeeding, pelvic floor, and psychosocial factors to postpartum sexual function; and (3) to identify latent trajectories and psychosocial risk markers for persistent dysfunction. We hypothesized that depressive symptoms and a negative body image would be more strongly associated with FSFI scores at 12 months postpartum than obstetric exposures (mode of birth, perineal pain, pelvic-floor symptoms), and that psychosocial variables would improve the discrimination of a prediction model for FSFI-defined dysfunction.

## 2. Materials and Methods

### 2.1. Study Design, Setting, and Ethics

We conducted a single-center, prospective cohort study at the Obstetrics and Gynecology Department and affiliated outpatient clinics of “Victor Babeș” University of Medicine and Pharmacy Timișoara. Consecutive pregnant adults attending routine antenatal care were approached between June 2024 and October 2024. This study followed participants from pregnancy through 12 months postpartum with standardized questionnaire batteries at predefined timepoints.

Eligibility criteria included age ≥ 18 years, singleton viable pregnancy, gestational age ≤ 20 weeks at enrollment, and sufficient Romanian language proficiency to understand and complete self-report instruments. Exclusion criteria were a severe psychiatric illness requiring acute treatment escalation, major fetal malformations diagnosed at enrollment, medically mandated abstinence from sexual activity at baseline, documented cognitive impairment, or anticipated relocation outside the catchment area before the postpartum visits. Missing data required exclusion from this study of the participant. The upper gestational-age limit of 20 weeks was chosen to ensure that baseline sexual-function assessments were collected before the onset of major third-trimester physical limitations and discomfort and to allow adequate time for women to contribute data at all three pregnancy trimesters.

The research protocol was reviewed and approved by the institutional Ethics Committee for the local scientific research ethics committee of the “Pius Brînzeu” (approval no. 445/13 April 2025) and adhered to the principles of the Declaration of Helsinki and good clinical practice in biomedical research. All participants provided written informed consent prior to any study procedures, including explicit consent for repeated contact and completion of intimate-sphere questionnaires. Personal identifiers were stored separately from study data, and all analyses used de-identified datasets. Personal identifiers (name, contact details) were stored in a password-protected file separate from de-identified study data, on secure institutional servers with access restricted to the core research team.

The primary outcome was the FSFI total score at 6–12 months postpartum (PP3), analyzed as a continuous variable. Secondary outcomes included FSFI-defined sexual dysfunction at PP3 under different thresholds, FSFI trajectories across six timepoints, and cluster-derived trajectory groups.

### 2.2. Participants, Follow-Up Schedule, and Procedures

At enrollment (first study visit during the first or early second trimester), eligible pregnant adults received brief verbal and written information outlining the study aims—understanding sexual function trajectories in pregnancy and postpartum—and the nature of the questionnaires. Those who consented completed baseline forms in a quiet room, without partners present, using paper or electronic formats according to preference. Trained research staff were available to clarify items but did not suggest specific responses. During the study period, 187 pregnant women met the criteria for longitudinal follow-up, of whom 90 were selected for having had FSFI values < 26.55.

Participants were followed at six timepoints: first trimester (T1, ≤13 + 6 weeks), second trimester (T2, 14–27 + 6 weeks), third trimester (T3, ≥28 weeks until delivery), and postpartum at 6–8 weeks (PP1), 3 months (PP2), and 6–12 months (PP3). FSFI was administered at all six timepoints. At PP3, additional instruments captured depressive symptoms, health-related quality of life, and body-image disturbance, and participants completed a structured questionnaire on breastfeeding patterns, pelvic-floor symptoms (urinary or bowel dysfunction), and perineal pain. PP3 visits were scheduled flexibly between 6 and 12 months postpartum to accommodate clinical and family commitments. For sensitivity analyses, PP3 assessments were dichotomized into ≤9 vs. >9 months postpartum.

Follow-up was coordinated with routine antenatal and postnatal visits when possible; otherwise, telephone reminders and flexible scheduling were used to reduce attrition. To minimize social desirability bias, partners were not present during completion of sexual function items, and participants were reminded that responses were confidential and would not influence their clinical care. Missing questionnaire items were checked at the point of collection, and participants were invited to complete omitted responses if they felt comfortable doing so.

### 2.3. Measures and Instruments

Sexual function was assessed with the Female Sexual Function Index (FSFI), a 19-item self-report instrument covering desire, arousal, lubrication, orgasm, satisfaction, and pain over the past four weeks. Items are scored on Likert-type scales, aggregated into domain scores (0–6) and a total score (2–36), with higher values indicating better sexual function. In this study, we used a culturally adapted Romanian version. Because FSFI cutoffs are derived in non-pregnant populations, we analyzed FSFI primarily as a continuous outcome, while using the widely cited threshold of 26.55 to define “FSFI-defined dysfunction” descriptively for postpartum comparisons.

At PP3, depressive symptoms were measured with the Patient Health Questionnaire-9 (PHQ-9), yielding a 0–27 total score, where higher scores indicate more severe depressive symptoms. Health-related quality of life was captured with the WHOQOL-BREF, which provides four domain scores—physical, psychological, social relationships, and environment—scaled 0–100, with higher scores reflecting a better perceived quality of life. We also administered a brief body-image disturbance scale (10 items, 5-point responses), summarizing into a 0–4 mean score where higher scores indicate greater dissatisfaction and self-consciousness regarding appearance during intimacy.

A structured questionnaire collected sociodemographic (age, education, marital status), anthropometric (self-reported pre-pregnancy weight and height to compute BMI), and obstetric data (parity, planned vs. unplanned pregnancy, gestational age at visits). Delivery details (mode of birth, use of episiotomy, instrumental delivery) were abstracted from medical records. Breastfeeding at PP3 was categorized as exclusive, partial, or none. Perineal pain at 6–8 weeks was rated on a 4-point scale and dichotomized into none/mild vs. moderate/severe for analysis. Pelvic-floor symptoms were defined as the presence of at least one of the following in the postpartum questionnaire: stress urinary incontinence, urge incontinence, fecal incontinence, or bothersome constipation.

Because FSFI cutoffs are derived in non-pregnant populations, we analyzed FSFI primarily as a continuous outcome. For secondary, descriptive purposes, we used the widely cited threshold of 26.55 to define ‘FSFI-defined dysfunction’ at PP3, while recognizing that this cutoff may overestimate clinically meaningful impairment in perinatal women [5,15]. To assess robustness, we conducted sensitivity analyses with alternative thresholds (FSFI < 24, approximating the lower quartile in our sample) and with domain-based definitions focusing on desire and pain subscales (e.g., desire score ≤ 3.0 or pain score ≤ 4.0 at PP3).

### 2.4. Statistical Analysis

All 150 women contributed FSFI data at all six timepoints (T1, T2, T3, PP1, PP2, PP3). Item-level missingness for psychosocial measures at PP3 was low (≤3% for PHQ-9, WHOQOL-BREF domains, body-image disturbance) and handled with complete-case analysis in multivariable and mediation models. Continuous variables were summarized as mean ± standard deviation (SD) and categorical variables as counts and percentages; normality was assessed with Shapiro–Wilk tests and inspection of histograms and Q–Q plots. FSFI trajectories were analyzed using repeated-measures ANOVA with the participant as the within-subject factor and Greenhouse–Geisser correction when sphericity was violated. As a sensitivity analysis, we fitted linear mixed-effects models with a random intercept for each participant and time (categorical) as a fixed effect, estimated by restricted maximum likelihood. Longitudinal FSFI patterns were explored using longitudinal k-means clustering on standardized FSFI total scores (T1–T3, PP1–PP3), evaluating k = 2–5 and selecting k = 3 based on the Calinski–Harabasz criterion (maximum at k = 3) and an average silhouette width of 0.53; 100 random starts and up to 500 iterations per start were used. Solutions with k = 2 and k = 4 were inspected as sensitivity analyses.

Postpartum sexual dysfunction was defined as FSFI < 26.55 at PP3 (6–12 months postpartum). Baseline and postpartum characteristics were compared between women with and without dysfunction using Welch’s *t*-tests for continuous variables and χ^2^ or Fisher’s exact tests for categorical variables. Pearson correlation coefficients quantified associations between FSFI at PP3 and PHQ-9, WHOQOL-BREF domains, body-image disturbance, age, and BMI. A multivariable linear regression model was constructed with PP3 FSFI as the dependent variable. Predictors were selected a priori based on a biopsychosocial framework and the prior literature [6,17,18,19,20,21]: exclusive breastfeeding (yes/no), moderate-to-severe perineal pain at PP1 (yes/no), pelvic-floor symptoms at PP3 (yes/no), primiparity (yes/no), and standardized (z-score) PHQ-9, WHOQOL-Psychological, body-image disturbance, and age.

An exploratory cross-sectional mediation analysis at PP3 examined whether PHQ-9 scores statistically mediated the association between body-image disturbance and FSFI, under standard linear-regression assumptions (linearity, no unmeasured confounding, no reverse causation), acknowledging that temporal precedence and causality cannot be inferred. For prediction of FSFI-defined dysfunction at PP3, logistic regression models were fitted with (a) PHQ-9 alone, (b) WHOQOL-Psychological alone, and (c) a combined psychosocial set (PHQ-9, WHOQOL-Psychological, body-image disturbance), with continuous predictors standardized (z-scores) and no interaction terms. Model performance was evaluated using AUC for discrimination; Brier score, calibration intercept and slope, and decile-based calibration plots for calibration; and internal validity by bootstrap resampling (1000 resamples).

## 3. Results

Women without dysfunction had a mean age of 28.4 ± 4.4 years and BMI of 24.7 ± 4.0 kg/m^2^, while those with dysfunction had corresponding values of 28.8 ± 4.2 years and 24.8 ± 3.8 kg/m^2^ (both *p* > 0.65). The proportion of primiparous women was modestly higher among those with dysfunction (45/90; 50.0%) than among those without dysfunction (26/60; 43.3%), but this difference was not statistically significant (*p* = 0.526). Planned pregnancies were somewhat more frequent in the dysfunction group (66.7% vs. 55.0%), suggesting a slightly different psychosocial profile, but here too the difference did not reach conventional significance (*p* = 0.204).

Obstetric and breastfeeding variables were not clearly associated with postpartum dysfunction. Cesarean delivery occurred in 29/60 (48.3%) women without dysfunction and in 36/90 (40.0%) women with dysfunction (*p* = 0.400). Exclusive breastfeeding at 6–12 months postpartum was reported by 33/60 (55.0%) women without dysfunction and 53/90 (58.9%) women with dysfunction (*p* = 0.762), indicating broadly comparable breastfeeding patterns across groups. In contrast, psychosocial measures showed more marked differences. Mean PHQ-9 scores were lower among women without dysfunction (6.7 ± 3.5) than among those with dysfunction (8.8 ± 3.3; *p* < 0.001), reflecting a higher burden of depressive symptoms in the latter. WHOQOL-Physical scores were higher in the no-dysfunction group (71.7 ± 12.2 vs. 67.1 ± 10.7; *p* = 0.019), as were WHOQOL-Psychological scores (72.6 ± 11.5 vs. 67.8 ± 11.4; *p* = 0.013). Body-image disturbance scores were slightly lower in women without dysfunction (1.4 ± 0.5) compared with those with dysfunction (1.6 ± 0.5), with a borderline *p*-value (*p* = 0.065), suggesting a possible trend toward more negative body image among those with poorer sexual outcomes.

When we used a more conservative cutoff of FSFI < 24 at PP3, the prevalence of dysfunction decreased to 44.0%, whereas a threshold of FSFI < 28 increased the prevalence to 72.0%. Across these alternative definitions—as well as domain-based definitions focusing on low desire (≤3.0) or pain (≤4.0)—women classified as having sexual dysfunction consistently had higher PHQ-9 scores, lower WHOQOL-Psychological scores, and greater body-image disturbance than women above the thresholds. The direction and magnitude of associations between psychosocial predictors and sexual dysfunction were similar to those observed with the FSFI < 26.55 cutoff, as presented in Table 1 and Table 2.

**Table 1:** Baseline and postpartum characteristics by sexual dysfunction at 6–12 months postpartum.

Variable	No Dysfunction (FSFI ≥ 26.55; n = 60)	Sexual Dysfunction (FSFI < 26.55; n = 90)	*p*-Value
Age, years	28.4 ± 4.4	28.8 ± 4.2	0.674
BMI, kg/m^2^	24.7 ± 4.0	24.8 ± 3.8	0.791
Primiparous, n (%)	26/60 (43.3%)	45/90 (50.0%)	0.526
Planned pregnancy, n (%)	33/60 (55.0%)	60/90 (66.7%)	0.204
Cesarean delivery, n (%)	29/60 (48.3%)	36/90 (40.0%)	0.4
Exclusive breastfeeding at 6–12 months, n (%)	33/60 (55.0%)	53/90 (58.9%)	0.762
PHQ-9 postpartum	6.7 ± 3.5	8.8 ± 3.3	<0.001
WHOQOL-Physical	71.7 ± 12.2	67.1 ± 10.7	0.019
WHOQOL-Psychological	72.6 ± 11.5	67.8 ± 11.4	0.013
Body-image disturbance score (0–4)	1.4 ± 0.5	1.6 ± 0.5	0.065

Tests: Welch’s *t*-test for continuous variables; χ^2^ test for categorical variables. BMI, body mass index; PHQ-9, Patient Health Questionnaire-9; WHOQOL, World Health Organization Quality of Life.

**Table 2:** Sensitivity analyses using alternative definitions of postpartum sexual dysfunction at 6–12 months (PP3).

Definition of Dysfunction at PP3	Prevalence n/N (%)	Mean PHQ-9 (±SD) in Dysfunction vs. No Dysfunction	Mean FSFI (±SD) in Dysfunction vs. No Dysfunction	Comment
FSFI total < 26.55 (main definition)	90/150 (60.0)	8.8 ± 3.3 vs. 6.7 ± 3.5	22.4 ± 2.5 vs. 29.6 ± 1.6	Primary classification used in main text
FSFI total < 24 (conservative)	66/150 (44.0)	9.1 ± 3.2 vs. 6.8 ± 3.4	21.0 ± 2.1 vs. 28.8 ± 2.3	Lower prevalence; stronger FSFI separation
FSFI total < 28 (liberal)	108/150 (72.0)	8.5 ± 3.3 vs. 6.2 ± 3.4	23.1 ± 2.7 vs. 30.2 ± 1.4	Highlights milder impairments
Desire domain ≤ 3.0	59/150 (39.3)	9.0 ± 3.2 vs. 6.8 ± 3.5	21.9 ± 2.3 vs. 28.9 ± 2.5	Focus on low desire
Pain domain ≤ 4.0	58/150 (38.7)	8.9 ± 3.3 vs. 6.9 ± 3.5	22.1 ± 2.4 vs. 28.7 ± 2.4	Focus on pain/dyspareunia

Across all definitions, higher PHQ-9 scores, lower WHOQOL-Psychological scores, and higher body-image disturbance were associated with dysfunction (all *p* < 0.01; ).

FSFI total scores displayed a marked trajectory across pregnancy and postpartum (Table 3). In the first trimester (T1), mean FSFI was 27.4 ± 3.9, consistent with generally preserved sexual function early in pregnancy. Scores declined modestly in the second trimester (T2, 25.4 ± 3.5) and more substantially in the third trimester (T3, 22.7 ± 4.2), reflecting progressive reductions in desire, comfort, and perhaps opportunity as pregnancy advanced. The most pronounced decline occurred in the early postpartum visit at 6–8 weeks (PP1), when the mean FSFI reached 20.1 ± 4.2, representing a drop of more than 7 points relative to T1. This postpartum nadir is compatible with a period of intense physical recovery, sleep deprivation, and adjustment to infant care.

Partial recovery was observed thereafter. At 3 months postpartum (PP2), the mean FSFI rose to 21.7 ± 3.9, suggesting some improvement as acute perineal discomfort abated and sexual relations were gradually resumed. By 6–12 months postpartum (PP3), the FSFI reached 25.5 ± 4.0, approaching but not fully matching first-trimester values. Despite this improvement, a substantial proportion of women remained below the FSFI dysfunction threshold at PP3, as noted above. Repeated-measures ANOVA confirmed a robust overall effect of the reproductive stage on FSFI (F = 124.1; *p* < 0.001).

In a sensitivity analysis stratifying the PP3 assessment by timing (≤9 vs. >9 months postpartum), the prevalence of FSFI-defined dysfunction was very similar (58.5% vs. 61.8%, *p* = 0.71). Mean FSFI at PP3 was 25.7 ± 4.0 in women assessed ≤9 months and 25.3 ± 4.1 in those assessed >9 months (mean difference 0.4 points; 95% CI −0.7 to 1.5; *p* = 0.48). A logistic regression model including PP3 timing (≤9 vs. >9 months) and the same psychosocial predictors as the main model showed no association between timing and dysfunction risk (adjusted odds ratio 1.12; 95% CI 0.60–2.10; *p* = 0.72), supporting the robustness of our findings across the 6–12-month window.

**Table 3:** FSFI total scores across pregnancy and postpartum.

Stage	FSFI Total (Mean ± SD)
First trimester (T1)	27.4 ± 3.9
Second trimester (T2)	25.4 ± 3.5
Third trimester (T3)	22.7 ± 4.2
6–8 weeks postpartum (PP1)	20.1 ± 4.2
3 months postpartum (PP2)	21.7 ± 3.9
6–12 months postpartum (PP3)	25.5 ± 4.0

Repeated-measures ANOVA: overall stage effect F(5745) = 124.1; *p* < 0.001. FSFI, Female Sexual Function Index; SD, standard deviation.

Table 4 presents FSFI totals at 6–8 weeks (PP1) and 6–12 months (PP3) postpartum for these subgroups. At PP1, women with vaginal birth and no or mild perineal pain had the highest FSFI (21.0 ± 3.8; n = 50). Women with cesarean birth had slightly lower scores (20.2 ± 4.8; n = 65), and those with vaginal birth and moderate/severe perineal pain had the lowest FSFI (18.6 ± 3.5; n = 35). One-way ANOVA indicated a statistically significant difference across groups at PP1 (F = 3.30; *p* = 0.040). By 6–12 months postpartum, group differences had largely attenuated. FSFI means converged to 25.8 ± 3.8 for vaginal birth without significant perineal pain, 24.6 ± 4.4 for vaginal birth with moderate/severe pain, and 25.8 ± 4.0 for cesarean birth. The overall ANOVA at PP3 was not significant (F = 1.22; *p* = 0.298).

FSFI total scores (mean ± SD, 95% CI) declined progressively from the first to the third trimester and only partially recovered postpartum. Specifically, FSFI total was 27.4 ± 3.9 (26.8–28.0) in the first trimester (T1), 25.4 ± 3.5 (24.8–26.0) in the second trimester (T2), and 22.7 ± 4.2 (22.0–23.4) in the third trimester (T3). Postpartum, sexual function remained impaired at PP1 with a mean FSFI of 20.1 ± 4.2 (19.4–20.8), showed slight improvement at PP2 to 21.7 ± 3.9 (21.1–22.3), and further increased by PP3 to 25.5 ± 4.0 (24.9–26.1), although it did not fully return to early-pregnancy levels.

Among the 150 participants, 86 (57.3%) reported exclusive breastfeeding at PP3, whereas 64 (42.7%) reported partial or no breastfeeding. Mean FSFI at PP3 was 25.9 ± 4.4 in the non-exclusive/none group and 25.3 ± 3.7 in the exclusive group (Table 5). Welch’s *t*-test revealed no significant difference (*p* = 0.403), suggesting that, in this cohort, exclusive breastfeeding per se was not a major determinant of sexual function at 6–12 months. Pelvic-floor symptoms were present in 55 women (36.7%) and absent in 95 (63.3%). At PP3, women without pelvic-floor symptoms had a mean FSFI of 25.7 ± 4.1, while those with symptoms had a mean of 25.2 ± 3.9 (*p* = 0.451). Thus, by 6–12 months postpartum, self-reported pelvic-floor dysfunction was not clearly associated with FSFI in this sample.

FSFI correlated moderately and inversely with depressive symptoms (PHQ-9, r = −0.39; *p* < 0.001), indicating that a higher depressive symptom burden was consistently associated with lower sexual function across desire, arousal, lubrication, orgasm, satisfaction, and pain domains. This correlation was among the largest observed, highlighting depressive symptoms as an important correlate of postpartum sexual health in this cohort. Conversely, FSFI correlated positively with WHOQOL-Psychological (r = 0.28; *p* = 0.001), suggesting that women who perceived better psychological quality of life also reported better sexual function. WHOQOL-Physical showed a weaker, borderline association (r = 0.16; *p* = 0.054), while WHOQOL-Social and WHOQOL-Environment were only minimally correlated with FSFI (r = 0.13, *p* = 0.120 and r = 0.03, *p* = 0.698, respectively). Body-image disturbance demonstrated a modest inverse correlation with FSFI (r = −0.24; *p* = 0.003), consistent with the notion that greater dissatisfaction and self-consciousness regarding one’s postpartum body may hamper sexual enjoyment. In contrast, age and BMI showed negligible correlations with FSFI (r = 0.02, *p* = 0.835 and r = −0.06, *p* = 0.475), suggesting that within the relatively narrow age and BMI ranges of this cohort, these anthropometric variables contributed little to variability in sexual function (Table 6).

The model included exclusive breastfeeding (yes/no), moderate-to-severe perineal pain (yes/no), pelvic-floor symptoms (yes/no), primiparity (yes/no), and standardized scores (z-scores) for PHQ-9, WHOQOL-Psychological, body-image disturbance, and age. The overall model explained approximately 19% of the variance in FSFI (R^2^ = 0.19; adjusted R^2^ = 0.15; F(8141) = 4.18; *p* < 0.001), indicating a modest but statistically significant combined predictive value.

Among the predictors, only depressive symptoms remained significantly associated with FSFI after adjustment. Each 1 SD increase in PHQ-9 was associated with a 1.2-point decrease in FSFI (β = −1.2; 95% CI −2.0 to −0.4; *p* = 0.005). WHOQOL-Psychological showed a positive but non-significant association (β = 0.5; 95% CI −0.2 to 1.2; *p* = 0.155), suggesting that its influence may overlap with that of depressive symptoms. Body-image disturbance, pelvic-floor symptoms, and age all showed small, non-significant coefficients. Primiparity had a negative coefficient (β = −1.2; 95% CI −2.4 to 0.0; *p* = 0.057), as can be seen in Table 7.

When predictors were standardized, the largest standardized coefficient was observed for PHQ-9 (β_std = −0.32), corresponding to a partial R^2^ of 0.10, whereas other predictors showed smaller partial R^2^ values (range 0.00–0.03). This supports depressive symptoms as an important, though not exclusive, correlate of postpartum sexual function.

The “resilient–high” cluster (33% of the sample) maintained comparatively high FSFI scores throughout: they started near 30.0 in T1, experienced only moderate declines in late pregnancy and early postpartum, and recovered to almost 29.0 by 6–12 months. Only about one in four women in this cluster met criteria for postpartum sexual dysfunction, suggesting a robust sexual resilience phenotype. The “recovering–moderate” group (42%) followed the global average more closely, with FSFI in the mid-20s early in pregnancy, more pronounced declines around childbirth, and partial recovery to 25.3 by PP3; nevertheless, postpartum dysfunction remained prevalent (60.3%). The “persistent–low” cluster (25%) showed low FSFI already in early pregnancy, marked deterioration toward the perinatal nadir, and only limited recovery by 6–12 months, with nearly 90.0% meeting dysfunction criteria (Table 8).

Table 9 provides a mediation analysis that moves beyond simple correlation to clarify mechanisms linking body-image disturbance and postpartum sexual function. The “a” path shows that for each 1-point increase in the body-image disturbance score (on its 0–4 scale), PHQ-9 increases by 2.4 points (95% CI 1.6–3.2; *p* < 0.001), indicating that poorer body image is strongly associated with more depressive symptoms. The “b” path demonstrates that each additional PHQ-9 point is associated with a 0.7-point decrease in FSFI (95% CI −0.9 to −0.5; *p* < 0.001). The total effect (“c” path) of body-image disturbance on FSFI is substantial and remains significant even after adjusting for PHQ-9 (direct effect “c”), indicating a pattern compatible with partial mediation. The bootstrapped indirect effect (−1.7; 95% CI −2.4 to −1.1; *p* < 0.001) confirms that a sizeable portion of the impact of body-image disturbance on sexual function operates through depressive symptoms. Because body-image disturbance, depressive symptoms, and FSFI were assessed concurrently at PP3, these results should be interpreted as cross-sectional mediation patterns rather than proof of causal pathways; longitudinal designs with repeated psychological assessments are needed to test directionality more rigorously.

Table 10 introduces a diagnostic–prognostic angle by quantifying how well psychosocial variables can discriminate which women will show FSFI-defined sexual dysfunction at 6–12 months postpartum. The PHQ-9-only model yields an AUC of 0.8 (95% CI 0.7–0.9), indicating good discrimination: a randomly chosen woman with dysfunction has roughly an 80.0% chance of having a higher predicted risk than a randomly chosen woman without dysfunction. At the optimal threshold (maximizing Youden’s index), this model achieves balanced sensitivity and specificity (both ≈ 0.7), with a PPV of 0.8 and NPV of 0.6, and a Brier score of 0.2, suggesting acceptable but not perfect calibration. The WHOQOL-Psychological-only model performs more modestly (AUC 0.7; sensitivity and specificity ≈ 0.6), indicating that perceived psychological quality of life alone is less powerful than depressive symptoms in classifying dysfunction. The combined psychosocial model, which includes PHQ-9, WHOQOL-Psychological, and body-image disturbance, improves the AUC to 0.9 (95% CI 0.8–0.9), with the sensitivity and specificity around 0.8 and a higher PPV (0.9) and NPV (0.7). The Brier score of 0.1 suggests better overall predictive accuracy and calibration.

Bootstrap internal validation yielded optimism-corrected AUCs of 0.78 for the PHQ-9-only model and 0.87 for the combined psychosocial model, with small increases in Brier scores (from 0.20 to 0.22 and from 0.10 to 0.12, respectively), indicating modest optimism in the apparent performance.

**Figure 1:**
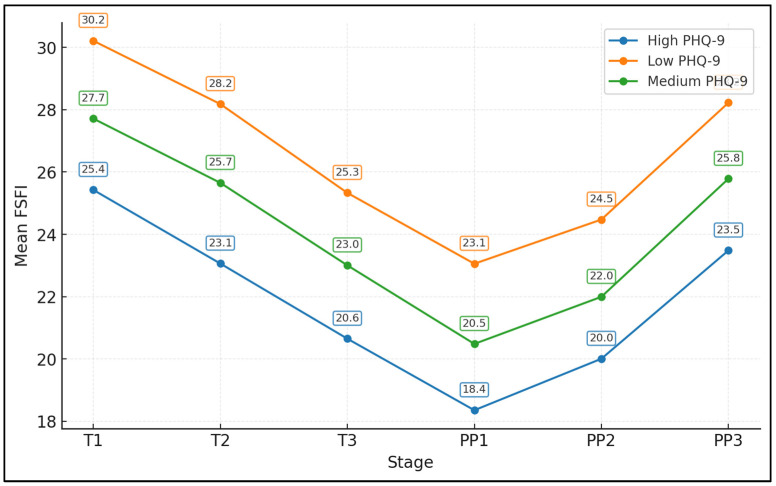
FSFI trajectories across pregnancy and postpartum by postpartum depressive-symptom tertiles. Women were grouped into tertiles based on PHQ-9 scores at 6–12 months postpartum: low (PHQ-9 ≤ 5; n = 51), medium (PHQ-9 6–9; n = 52), and high (PHQ-9 ≥ 10; n = 47). Lines depict mean FSFI at each timepoint (T1–T3, PP1–PP3) within each tertile group, with error bars representing standard errors.

Figure 1 shows mean FSFI scores at each of the six timepoints for women grouped by postpartum PHQ-9 tertiles (low, medium, high). As shown here, all 150 enrolled women contributed FSFI data at each of the six timepoints (T1–T3, PP1–PP3), yielding 900 person-time observations for trajectory analyses. Women with low PHQ-9 scores had the highest FSFI at every stage, from 30.2 in the first trimester to 28.2 at 6–12 months postpartum. The medium PHQ-9 group showed intermediate values (e.g., 27.7 at T1 and 25.8 at PP3), while the high PHQ-9 group had a consistently lower FSFI (25.4 at T1 and 23.5 at PP3). All groups exhibited a similar shape: a decline from T1 to a nadir at 6–8 weeks postpartum (PP1), where FSFI reached 23.1, 20.5, and 18.3 in the low, medium, and high PHQ-9 groups, respectively, followed by partial recovery toward PP3. The roughly 4.7-point gap in FSFI between low and high PHQ-9 groups at PP3 (28.2 vs. 23.5) illustrates a clinically relevant separation, reinforcing depressive symptoms as a key stratifier of both the depth of the postpartum nadir and the degree of recovery in sexual function.

**Figure 2:**
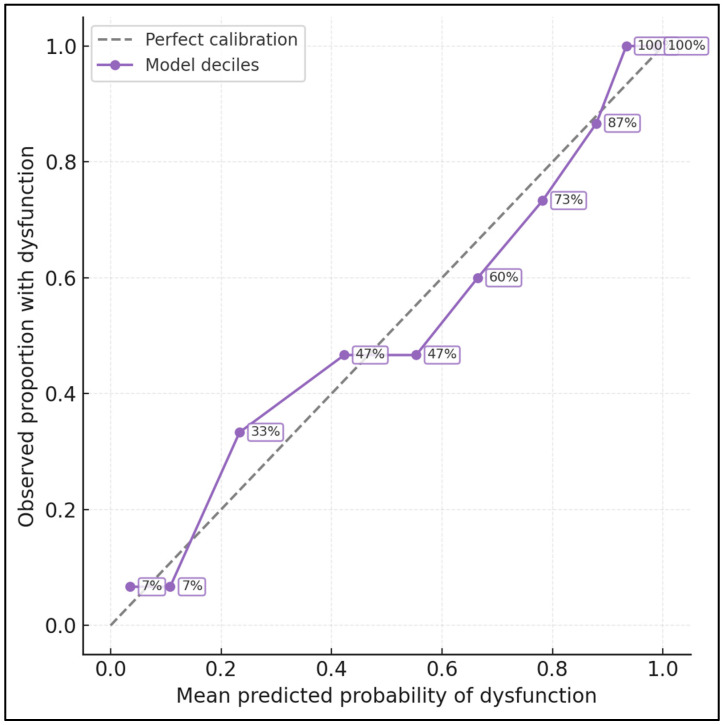
Calibration of the psychosocial prediction model for postpartum sexual dysfunction at 6–12 months. Women were divided into deciles of predicted risk based on a logistic regression model including PHQ-9, WHOQOL-Psychological, and body-image disturbance. The x-axis shows the mean predicted probability of FSFI-defined dysfunction within each decile, and the y-axis shows the corresponding observed proportion with FSFI < 26.55. Circles represent observed vs. predicted values for each decile, and the solid curve connects these points to visualize calibration. The dashed 45° line represents perfect calibration (predicted = observed).

Figure 2 evaluates how well a logistic model using PHQ-9, WHOQOL-Psychological, and body-image disturbance predicts FSFI-defined dysfunction at 6–12 months postpartum. Women were divided into deciles of predicted risk; for each decile, the mean predicted probability of dysfunction is plotted against the observed proportion with FSFI < 26.55. In the lowest-risk decile, the model predicted a dysfunction probability of 3.5%, while the observed rate was 6.7%. At intermediate deciles, predicted and observed risks were closely aligned (e.g., in the 6th decile, 66.5% predicted vs. 60.0% observed), and in the highest deciles, both approached 100% (predicted 98.7% vs. observed 100% in the top decile). The decile curve tracks the 45° reference line reasonably well, with slight underprediction around the middle of the distribution and near-perfect calibration at the extremes. This figure illustrates that a simple psychosocial model can not only discriminate high- vs. low-risk women but also produce well-calibrated absolute risk estimates suitable for clinical risk stratification. Calibration-in-the-large for the combined psychosocial model was close to ideal (intercept −0.04; 95% CI −0.19 to 0.11), and the calibration slope was 0.96 (95% CI 0.75–1.17), indicating only mild over- or under-fitting.

## 4. Discussion

### 4.1. Summary of Evidence

In this cohort, depressive symptoms, body-image disturbance, and psychological quality of life showed stronger associations with postpartum sexual function than mode of birth, breastfeeding, or a broad dichotomous measure of pelvic-floor symptoms. Our findings remain consistent with data from large observational series. Classic work from the UK and the Netherlands showed that difficulties with desire, arousal, and dyspareunia remain common up to one year after childbirth, even though there is partial recovery from the early postpartum nadir [16,17]. Khajehei et al. reported that 64% of Australian women scored in the range of sexual dysfunction at around six months postpartum, very similar to the proportion we observed slightly later in the first postpartum year [18]. A recent systematic review and meta-analysis by Cattani et al. synthesized 30 studies and found that the prevalence may reach 83% at three months and 64% at six months postpartum, depending on the instrument and timing, reinforcing the notion that sexual difficulties are the rule rather than the exception in the first year after birth [19]. Our longitudinal FSFI trajectory—with a marked decline from late pregnancy to 6–8 weeks, followed by incomplete recovery by 6–12 months—therefore mirrors the general pattern described in these cohorts, while our cluster analysis adds nuance by showing distinct “resilient–high”, “recovering–moderate”, and “persistent–low” subgroups rather than a uniform trajectory.

The magnitude of change in FSFI across the perinatal period is also clinically meaningful. A decline from 27.4 in early pregnancy to 20.1 at 6–8 weeks postpartum represents a shift from well-preserved function to a profile characterized by low desire, reduced arousal and lubrication, and frequent pain or discomfort during intercourse. In our cohort, many women in this range also fell below the widely used dysfunction threshold of 26.55 and reported concomitant dyspareunia, vaginal dryness, and diminished satisfaction. Even though FSFI lacks a universally accepted minimal clinically important difference in perinatal populations, the observed seven-point drop is likely to reflect a transition from relatively satisfying sexual activity to more frequent avoidance, distress, or compromise, which aligns with qualitative descriptions from postpartum women in other studies [16,17,18].

With respect to obstetric determinants, our data suggest that the mode of birth and perineal pain mainly shape early postpartum sexual function, with differences largely attenuating by 6–12 months. This is broadly aligned with earlier cohort studies in which women with instrumental birth or more severe perineal trauma reported more dyspareunia and sexual difficulties in the first months after delivery, but group differences diminished over time [16,17]. The meta-analysis by Cattani et al. similarly concluded that perineal trauma (especially obstetric anal sphincter injury and episiotomy) increases the odds of sexual dysfunction and dyspareunia in the first postpartum year, whereas a caesarean section reduces the dyspareunia risk but not the overall sexual dysfunction risk [19]. More recent prospective data from Gommesen et al. show that higher-degree perineal tears and anal sphincter injury are strongly associated with dyspareunia at 12 months, even though many women remain sexually active and report otherwise acceptable function [22]. Our finding that moderate/severe perineal pain was associated with a lower FSFI shortly after birth but not at 6–12 months is consistent with these results, suggesting that the physical sequelae of perineal trauma are most salient for short- to medium-term pain and penetrative comfort, whereas longer-term global sexual function depends increasingly on psychological and relational factors.

In contrast, exclusive breastfeeding and a broad dichotomous measure of pelvic-floor symptoms showed little association with the FSFI at 6–12 months in our cohort. Khajehei et al. observed that breastfeeding was related to more sexual problems and lower FSFI scores early in the postpartum period, but the effect was attenuated at later follow-up, and other predictors such as fatigue, relationship dissatisfaction, and depressive symptoms became more prominent [18]. Cattani et al. likewise reported mixed evidence for breastfeeding as a risk factor: some studies found transient associations with dyspareunia and vaginal dryness in the hypoestrogenic, amenorrheic months, while others did not confirm a clinically relevant long-term effect [19]. Regarding pelvic-floor function, longitudinal work in Europe and Australasia has linked severe urinary or anal incontinence to greater sexual distress and coital incontinence, but the impact on overall sexual function scores is often modest and mediated by self-perceived health and partner support [17,22]. Our null findings for pelvic-floor symptoms may therefore reflect limited power, use of a simple present/absent composite, and the relatively late postpartum timepoint; more granular measures of incontinence severity, coital leakage, and pelvic pain might still reveal associations in subgroups.

Psychological determinants emerged as the most powerful correlates of postpartum sexual function in our data, with depressive symptoms showing the largest standardized association and retaining significance in multivariable models. This is congruent with the pilot study by Chivers et al., who found that postpartum women with elevated Edinburgh Postnatal Depression Scale scores had substantially lower FSFI totals and lower arousal, orgasm, and satisfaction subscale scores than non-depressed women, despite similar exposure to obstetric risk factors [20]. Our mediation analysis, in which postpartum depressive symptoms partially explained the link between body-image disturbance and lower FSFI, dovetails with the findings of Ozcan, who reported that a poorer postpartum body image in primiparous women was moderately correlated with higher depression and fatigue and inversely correlated with sexual function (r ≈ −0.37) [21]. Together, these results support a biopsychosocial model in which negative body image, fatigue, and mood symptoms form an interconnected cluster that erodes desire, arousal, and satisfaction, particularly in women who already follow a “persistent–low” sexual trajectory. They also highlight the importance of looking beyond obstetric variables when counseling women about their sexual prognosis after childbirth.

From a clinical and public health perspective, our risk-prediction findings suggest a feasible pathway to more proactive screening and targeted intervention. The good discrimination of PHQ-9 alone, and excellent discrimination of the combined psychosocial model, indicates that standard mental health and quality-of-life instruments already used in perinatal care can help identify women at high risk of persistent dysfunction. This dovetails with the emerging intervention literature. A recent systematic review and meta-analysis of nonpharmacological treatments for postpartum sexual dysfunction concluded that evidence for Kegel exercises, pelvic-floor muscle training, and structured counseling remains heterogeneous and often of low quality, with modest average effect sizes but wide confidence intervals, underscoring the need to better target those most likely to benefit [23]. More recently, Mao et al. showed in a randomized trial that adding pelvic myofascial trigger-point release to structured pelvic-floor training significantly improved FSFI scores, pelvic-floor electromyography parameters, and pain outcomes in women with sexual dysfunction after vaginal delivery, compared with pelvic-floor training alone [24].

Our observation that depressive symptoms at 6–12 months postpartum are closely linked to sexual dysfunction is consistent with broader work on perinatal mood trajectories, in which early ‘baby blues’ or maternity blues phenomena mark a subgroup at increased risk of persistent depressive symptomatology [25,26]. Longitudinal studies have shown that transient early mood disturbance can evolve into full-blown perinatal depression in a sizable minority of women [25] and that higher maternity blues scores are associated with more severe anhedonia, anxiety, and depression components on the Edinburgh Postnatal Depression Scale [26]. Incorporating brief tools that screen for both early postpartum mood lability and more sustained depressive symptoms may therefore help clinicians identify women at risk of both mental health and sexual health sequelae.

In practical terms, our data suggest that women with PHQ-9 scores ≥ 10 and body-image disturbance scores ≥ 2.0 at 6–8 weeks postpartum fall disproportionately into ‘persistent–low’ FSFI trajectories and have a high predicted probability of dysfunction at 6–12 months (apparent predicted risk ≥ 0.70 in our sample). Such thresholds could be used as a simple screening rule-of-thumb to prompt more detailed assessment and early referral. However, because our prediction model was derived and internally validated within the same cohort, these cutoffs should be regarded as provisional and subject to refinement once external validation data become available.

These findings support a routine, structured discussion of sexual health throughout pregnancy and the first postpartum year, rather than limiting counseling to the 6-week check. Because depressive symptoms and body-image disturbance were more strongly related to postpartum sexual outcomes than the mode of birth, breastfeeding status, or simple pelvic-floor symptom screens, integration of brief mental health (PHQ-9) and body-image assessments into perinatal care could help identify the women at greatest risk. The good discrimination of the combined psychosocial model (AUC 0.9) indicates that existing tools could underpin simple risk-stratification algorithms, guiding stepped care: low-intensity psychoeducation for lower-risk women and early referral to perinatal mental health, pelvic floor, or sex therapy services for those with a high predicted risk or “persistent–low” sexual trajectories. Embedding partner-inclusive counseling, normalizing fluctuating sexual function, and clarifying realistic expectations about recovery may further reduce distress and support relationship stability.

### 4.2. Limitations

This single-center cohort from an urban academic tertiary hospital in western Romania may not fully represent women receiving care in rural settings, non-academic facilities, or health systems with different perinatal pathways and cultural norms regarding sexuality. Eastern European contexts, including Romania, may differ from Western Europe or North America in terms of routine postpartum follow-up, access to pelvic floor and mental health services, and openness in discussing sexual concerns. These factors limit external generalizability and highlight the need for replication in more diverse geographic and healthcare settings. The sample size, while adequate for the planned analyses, restricts power to explore interactions and finer subgroup effects (specific pelvic-floor disorders or detailed breastfeeding trajectories). All key determinants were captured through self-report instruments, which are vulnerable to recall and social desirability biases, particularly for intimate topics. The FSFI cutoff of 26.55 was derived in non-pregnant populations and may not perfectly reflect clinically meaningful dysfunction in the perinatal context. Although we modeled multiple psychosocial pathways, unmeasured factors—such as relationship satisfaction, partner sexual function, sexual self-schema, history of sexual trauma, and hormonal biomarkers—were not assessed and may confound or moderate the observed associations. The modest explained variance of the multivariable model (R^2^ = 0.19) should therefore be interpreted as indicating that depressive symptoms and body-image disturbance account for a meaningful but limited share of postpartum sexual function variability, with substantial room for future models to incorporate dyadic, relational, and biological dimensions. In particular, we did not assess partner-related variables such as relationship quality, partner mental health, and partner sexual function, which are likely to shape postpartum sexual trajectories and could improve both explanatory and predictive models in future dyadic studies. Furthermore, the predictive models were developed and evaluated in the same dataset. Although bootstrap internal validation suggests only modest optimism, external validation in independent cohorts is essential before these tools can be recommended for routine clinical risk stratification. Future studies should test the model in diverse populations, recalibrate intercepts and slopes where necessary, and explore whether adding relationship-level variables or obstetric details improves discrimination and calibration. Finally, the multivariable and prediction models were developed and evaluated in the same dataset, introducing a risk of optimism, so external validation in independent cohorts is needed before clinical implementation.

## 5. Conclusions

In this longitudinal cohort followed from early pregnancy to 12 months postpartum, sexual function showed a clear pattern of decline toward an early postpartum nadir, followed by only partial recovery. Obstetric and breastfeeding variables had limited long-term impact, whereas depressive symptoms, body-image disturbance, and psychological quality of life emerged as central correlates and predictors of postpartum sexual outcomes. Distinct FSFI trajectory clusters and a highly discriminative psychosocial prediction model underline the heterogeneity of postpartum sexual adaptation and the feasibility of targeted risk stratification using tools already familiar to perinatal care providers. Future work should validate these findings in broader populations, incorporate partner and relational measures, and test integrated interventions that combine pelvic-floor rehabilitation with mental health and body-image-focused support to modify high-risk trajectories and improve sexual well-being after childbirth.

## Figures and Tables

**Table 4:** FSFI total by mode of birth and perineal pain.

Group	n	FSFI PP1 (6–8 Weeks)	FSFI PP3 (6–12 Months)
Vaginal, no/moderate perineal pain	50	21.0 ± 3.8	25.8 ± 3.8
Vaginal, moderate/severe perineal pain	35	18.6 ± 3.5	24.6 ± 4.4
Cesarean	65	20.2 ± 4.8	25.8 ± 4.0

ANOVA: PP1 F = 3.30, *p* = 0.040; PP3 F = 1.22, *p* = 0.298. FSFI, Female Sexual Function Index; PP, postpartum.

**Table 5:** FSFI at 6–12 months postpartum by breastfeeding and pelvic-floor symptoms.

Group	n	FSFI PP3 (Mean ± SD)	*p*-Value
Non-exclusive or no breastfeeding	64	25.9 ± 4.4	0.403
Exclusive breastfeeding	86	25.3 ± 3.7	–
No pelvic-floor symptoms	95	25.7 ± 4.1	0.451
Pelvic-floor symptoms present	55	25.2 ± 3.9	–

Tests: Welch’s *t*-tests comparing exclusive vs. non-exclusive breastfeeding and pelvic-floor symptoms vs. none. FSFI, Female Sexual Function Index; PP3, 6–12 months postpartum.

**Table 6:** Correlates of FSFI at 6–12 months postpartum.

Predictor	r	*p*-Value
PHQ-9 postpartum	−0.39	<0.001
WHOQOL-Physical	0.16	0.054
WHOQOL-Psychological	0.28	0.001
WHOQOL-Social	0.13	0.12
WHOQOL-Environment	0.03	0.698
Body-image disturbance score (0–4)	−0.24	0.003
Age, years	0.02	0.835
BMI, kg/m^2^	−0.06	0.475

Test: Pearson correlation. PHQ-9, Patient Health Questionnaire-9; WHOQOL, World Health Organization Quality of Life; BMI, body mass index.

**Table 7:** Multivariable predictors of FSFI at 6–12 months postpartum (linear regression).

Predictor	β (Unstandardized)	95% CI	*p*-Value
Exclusive breastfeeding (yes vs. no)	−0.7	−1.9 to 0.6	0.278
Moderate/severe perineal pain (yes vs. no)	0	−1.6 to 1.7	0.953
PHQ-9 postpartum (per SD)	−1.2	−2.0 to −0.4	0.005
WHOQOL-Psychological (per SD)	0.5	−0.2 to 1.2	0.155
Body-image disturbance (per SD)	−0.2	−1.1 to 0.6	0.56
Pelvic-floor symptoms (yes vs. no)	−0.3	−1.5 to 1.0	0.666
Age (per SD)	−0.0	−0.7 to 0.6	0.879
Primiparous (yes vs. no)	−1.2	−2.4 to 0.0	0.057

Model: R^2^ = 0.19; adjusted R^2^ = 0.15; F(8141) = 4.18; *p* < 0.001. Continuous predictors (PHQ-9, WHOQOL-Psychological, body-image disturbance, age) were standardized (z-scores). PHQ-9, Patient Health Questionnaire-9; WHOQOL, World Health Organization Quality of Life; SD, standard deviation; CI, confidence interval.

**Table 8:** Latent FSFI trajectory clusters identified by longitudinal k-means.

Cluster (Label)	n	FSFI T1	FSFI T2	FSFI T3	FSFI PP1	FSFI PP2	FSFI PP3	Postpartum Dysfunction at PP3 (FSFI < 26.55), n (%)
1—Resilient–high	49	29.9	28.3	25.7	23.1	24.7	28.9	14 (28.6%)
2—Recovering–moderate	63	27.3	25.2	22.4	19.6	21.4	25.3	38 (60.3%)
3—Persistent–low	38	23.7	22.1	18.9	16.8	18.3	21.7	34 (89.7%)

FSFI, Female Sexual Function Index; T1–T3, pregnancy trimesters; PP1–PP3, postpartum visits at 6–8 weeks, 3 months, and 6–12 months. Clusters derived from k-means on standardized FSFI across six timepoints (k = 3, Calinski–Harabasz criterion). Mixed-effects model with time × cluster interaction *p* < 0.001.

**Table 9:** Mediation of the association between body-image disturbance and postpartum FSFI by depressive symptoms.

Path/Effect	Dependent Variable	Predictor	B (Unstandardized)	95% CI	*p*-Value
a (body-image → PHQ-9)	PHQ-9	Body-image score (per 1-point)	2.4	1.6 to 3.2	<0.001
b (PHQ-9 → FSFI)	FSFI	PHQ-9 (per 1-point)	−0.7	−0.9 to −0.5	<0.001
c (total effect)	FSFI	Body-image score (per 1-point)	−2.9	−3.9 to −1.9	<0.001
c′ (direct effect)	FSFI	Body-image score (per 1-point), adjusted for PHQ-9	−1.2	−2.1 to −0.3	0.009
Indirect effect (a × b)	FSFI	Body-image via PHQ-9	−1.7	−2.4 to −1.1	<0.001

Linear regressions adjusted for age, BMI, primiparity, and mode of birth. Indirect effect estimated via non-parametric bootstrap (5000 resamples, bias-corrected confidence intervals). Model R^2^ for FSFI with both predictors ≈ 0.3.

**Table 10:** Discrimination of postpartum FSFI-defined sexual dysfunction by psychosocial models.

Model	AUC	95% CI	Sensitivity	Specificity	PPV	NPV	Brier Score
PHQ-9 only	0.8	0.7 to 0.9	0.7	0.7	0.8	0.6	0.2
WHOQOL-Psychological only	0.7	0.6 to 0.8	0.6	0.6	0.7	0.4	0.2
Combined psychosocial model (PHQ-9 + WHOQOL-Psychological + body-image)	0.9	0.8 to 0.9	0.8	0.8	0.9	0.7	0.1

Outcome: FSFI-defined sexual dysfunction at 6–12 months postpartum (FSFI < 26.55). AUC, area under the receiver operating characteristic curve. PPV, positive predictive value; NPV, negative predictive value. Predictions obtained from logistic regression models; Brier score quantifies overall prediction error (lower is better). Metrics calculated at thresholds maximizing Youden’s index.

## Data Availability

The original contributions presented in this study are included in the article. Further inquiries can be directed to the corresponding author.
